# Lectures on the Diagnosis and Treatment of Diseases of the Lungs

**Published:** 1841-06-05

**Authors:** W. W. Gerhard


### MEDICAL EXAMINER.

DEVOTED TO MEDICINE, SURGERY, AND THE COLLATERAL SCIENCES.

No. 23.] PHILADELPHIA, SATURDAY, JUNE 5, 1841. [Vol. IV.

### LECTURES ON THE DIAGNOSIS AND TREATMENT OF DISEASES OF THE LUNGS.

BY W. W. GERHARD, M. D.

### LECTURE XIII.—(*Continued.*)

#### PHTHISIS—TREATMENT.

There are several remedies which at one time enjoyed a reputation in the treatment of consumption, which is by no means merited. The principal of these are digitalis, hydrocyanic acid, and the acetate of lead. Digitalis was given mainly because the excessive frequency of the pulse, which constitutes so prominent a symptom in many cases of phthisis, cannot be reduced by blood-letting, while it will sometimes partially yield to digitalis. The powers of this remedy extend no further; and as the good results of it are extremely doubtful, while it is often positively mischievous, it is now almost abandoned. I have from time to time made a trial of its virtues, but without satisfactory results.

Much was expected from hydrocyanic acid when it first came into use. It is certainly a good sedative; but as the remedy is necessarily extremely uncertain, and is attended with no little danger when the strength of it happens to be greater than usual, it is, in fact, not much prescribed. I do not, however, object to it, as it unquestionably is a good anodyne. The ferrocyanate of potassa is occasionally a good remedy, possessing some sedative powers, and to some extent controlling the night sweats; the dose which I prefer is five grains three or four times daily, gradually increased to twice that amount, should no effect follow. One of the advantages of the wild cherry bark certainly arises from the proportion of prussic acid which it contains; this is sometimes considerable enough to cause some fever, and a disagreeable sensation of feebleness in the head.

The acetate of lead I have rarely used in the treatment of phthisis proper, although it is recommended for its power in checking the hectic fever. But in the diarrhœa which often occurs, it is one of the most useful remedies, given in combination with a small dose of opium.

The chalybeates are occasionally resorted to in the treatment of phthisis, especially the iodide of iron, which is given in doses of from ten to thirty drops of the solution two or three times daily. Few patients will bear larger doses, which are apt to cause nausea and a disagreeable feeling of constriction at the epigastrium and head. My own impressions are less favourable to this remedy than they formerly were; it certainly acts well in some cases, but fails entirely as a curative agent, and from its aptness to cause the disagreeable symptoms just referred to, it cannot be given in as large doses, or as frequently as would otherwise be desirable. It differs a little from other chalybeates, and possesses more decidedly alterative properties. Other forms of the same class of remedies are occasionally resorted to; and, like the preparations of iodine, answer best when the patient is depressed, with little or no febrile excitement, and the constitution is feeble and deteriorated.

There are certain mineral waters which have acquired more or less celebrity in the treatment of phthisis: amongst these are some of the springs in the Pyrenees, and the Red Sulphur Springs in Virginia. These are all situated in mountainous districts, which renders the climate injurious to many classes of phthisical patients. In advanced cases, the benefit, if any result, can be but palliative; and the circumstances attending the position of the springs, and the long journey necessary to reach them, should be taken into the account, before advising patients to resort to them. My own experience as to their virtues is limited to a short residence at the Red Sulphur. This is a cold and very agreeable water, containing very little saline substance, and impregnated with a moderate quantity of sulphuretted hydrogen. The analysis of the spring is found, however, not to be complete enough to render it conclusive. The most benefit was derived by patients who were in need of an alterative, especially of one which was capable of acting upon the digestive canal. Such cases appeared to derive essential benefit from the combined influence of the water and the journey; and in this way, at least, it appears to be serviceable in commencing phthisis, where the irritability of the chest is not great, and there is little or no tendency to acute bronchitis or pleurisy. In the latter cases the climate does not appear to me very favorable, at least not in a wet summer.

The treatment, therefore, of phthisis, is almost entirely indirect, and we hope to check the progress of tubercles by removing accidental complications, or diseases and conditions of body which favour their growth, rather than by acting directly upon the tuberculous secretion. Hence it is necessarily uncertain, and often fails when every thing seems to be most promising; for as tubercles themselves are manifested by but few symptoms, the greatest part of the sufferings of the patient is caused by the complications. These are often readily removed, and the patient is apt to fancy that the apparent amelioration is real.

### PNEUMOTHORAX.

There is a lesion of the lungs and pleuræ which is rather a result of disease than a positive morbid action. This is pneumothorax, or perforation of the lung. It is true that as soon as this accident occurs, pleurisy is set up, and only differs from common inflammation in the mingling of the symptoms of the pleurisy with those of the perforation. The mechanism of perforation is very simple; in almost every case it results from tuberculous disease of the lungs, but any alteration of those organs situated near the pleura, and gradually destroying the parenchyma beneath it, may produce the same result. As soon as the pleura is left unsupported by the tissue of the lungs, it becomes of a dull yellow colour, and soon sloughs; a small hole forms in the centre of the dead portion, which is enlarged by the passage of air through it during the act of inspiration. The size of this opening varies from that of a pin's head to a third of an inch in diameter; it is generally of a valvular form, and allows with difficulty the passage of the air from the pleura. As the air enters more easily than it passes out, it of course accumulates in the cavity, and the chest quickly increases in volume, from the quantity of atmospheric air which finds its way into it.

The air is an immediate irritant to the serous membrane, and gives rise to inflammation, which is followed by the secretion of its usual products, lymph and serum. The latter accumulates at the bottom of the cavity, mixed with a few flocculi of lymph, but the greater part of this substance adheres to the surface of the serous coat in the form of a false membrane, which extends to the point of perforation, and then closes it completely, in which case there is no difference between empyema and advanced pneumothorax. The liquid contained in the pleura is at first merely serum, but it afterwards is replaced by pus, which is secreted from the false membrane as in chronic pleurisy.

As pneumothorax is a physical lesion which produces a rapid change in the condition and functions of the viscera of the chest, its physical signs are very evident, and are often beautiful illustrations of the accuracy of physical exploration. The immediate result of the passage of the air into the cavity of the pleura is the collapse of the lung; the inspiratory murmur therefore ceases or is replaced by amphoric respiration, which is often heard over the whole cavity, and in other cases is limited to the part nearest the perforation; the sign is much clearer and sharper than in those cases in which it is caused by a cavity in the substance of the lung, for the walls of the chest are more elastic and produce a clearer sound than those of an ordinary cavity. The expiration, however, is often unheard, for the opening is in many cases too small to allow the air to pass with sufficient freedom to give much sound. In this respect the amphoric respiration differs from that of pulmonary cavities.

The amphoric respiration often ceases after the pus has increased, and the coating of lymph has formed over the opening, and there is then either no sound, or a slight and bronchial respiration heard at a distance at the root of the lungs.

As a necessary attendant upon the amphoric respiration, we find a corresponding resonance of the voice, which follows the same course, and ceases at the same time. The metallic tinkling is another phenomenon of equal interest. It resembles the tinkling of a pin against the sides of a glass or metallic vessel more nearly than any thing else, and was at one time supposed to depend upon the dropping of a small portion of liquid from the top of the pleura upon the surface of the effusion. Dr. Bigelow, of Boston, performed a number of experiments upon the dead body, and satisfied himself that the cause of the tinkling depended upon the air forcing its way upwards through the liquid, and not in the dropping from above. The tinkling is by no means a constant sign, and is, therefore, much less important than the amphoric respiration and resonance of the voice.

The signs of pneumothorax gradually decline as it passes into ordinary empyema, and the ordinary flatness of percussion follows, with entire absence of respiratory murmur. The quantity of pus is much greater than in common cases of pleurisy; it sometimes amounts to several gallons, and causes extreme difficulty of the respiration.

The rational symptoms of pneumothorax are by no means conclusive, but in most cases they are sufficiently well marked to excite a suspicion of the nature of the accident. They are the usual signs of acute pleurisy with extreme and sudden dyspnœa from the rapid entrance of air into the cavity of the pleura. Their uncertainty arises from the occasional absence of pain in cases of acute pleurisy, and from the dyspnœa not being always very intense. As a general rule, however, if a patient labouring under symptoms of phthisis be taken with very sudden and acute pain in the chest and extreme dyspnœa, there is strong reason for suspecting that perforation of the pleura has taken place; especially if the pain occur during an effort of coughing, or some other sudden shock given to the chest. It is true that all of these symptoms may depend on acute secondary pleurisy, which sometimes developes itself, or at least shows itself almost instantaneously, and the test is therefore to be sought in the physical signs of the disease, which are alone to be relied upon. The pain is described as similar to that occurring in severe cases of pleurisy, as cutting or lancinating, and at first prevents the patient from lying on the affected side, but after the disease has continued for a time, the patient follows the ordinary rule of chronic pleurisy, and lies on his back or on the affected side, in order to avoid the presence of a large quantity of liquid upon the mediastinum. The other symptoms are also those of pleurisy; the fever which follows the perforation is of the acute kind observed in cases of pleurisy, with a rapid and rather wiry pulse, followed by abundant sweats at night. After the effusion has become purulent, the fever approaches more nearly to the hectic form, and the patient complains much more frequently of chills than he does in the earlier stages of the disease. The patient gradually loses flesh, but does not become nearly as much emaciated as in those cases in which tuberculous disease is passing through its ordinary course; nor is the disturbance of his general health nearly as great, provided he escape the first dangers of the accident. The other functions of the body are more or less disordered, but in very different degrees, and are scarcely similar in two patients. This variety depends upon the different susceptibility of individuals, which necessarily renders all the accidental or secondary symptoms of a local inflammation extremely uncertain and variable, nor can they be described except in general terms, and as innowise necessary to characterize the affection. The symptoms of the original disease causing the pneumothorax, in great part remain, but are in some degree modified by it; thus the cough and expectoration diminish when perforation supervenes, for the difficulty of breathing and pain prevent a full expiration, which is necessary to a complete cough; the cough which is proper to pneumothorax is even shorter and drier than that of pleurisy, for the respiration is less complete and more painful. Of course no expectoration can arise from the pneumothorax, if there be any; it must depend upon accompanying disease of the lungs or bronchial tubes.

*Diagnosis and Prognosis.*—The diagnosis of pneumothorax, since the discovery of physical exploration, is amongst the most certain of those of diseases of the chest, for in a lesion of this kind the physical signs are pathognomonic; but without them, the lesion may be suspected, but cannot be certainly recognised or distinguished from acute pleurisy. Physical exploration goes much farther than the mere recognition of the disease; it points out its different degrees and stages, and the gradual passage of it into empyema. The prognosis is more uncertain; in the large majority of cases it is unfavourable, and speedily fatal; but this rapid termination depends less on the lesion itself than upon the disease which has given rise to it, or on the combined influence of the two; If, for instance, one lung be almost unfitted for respiration, and the perforation happen in that which is comparatively healthy, respiration is almost interrupted, for both lungs are rendered nearly useless, and the patient dies in a few hours or days from exhaustion and orthopnœa: hence the condition of the lung which is not the seat of the perforation has much influence upon the prognosis. If the patient does not labour under any immediate danger from the interruption to the respiration, the prognosis is still almost necessarily fatal if the phthisis be at all advanced; but if it be confined to a few scattered tubercles, it has little influence upon the course of the pneumothorax, which seems rather to retard than hasten the progress of tubercles. If the disease assume the latter form and arise merely from the accidental rupture of a small tubercle into the pleuræ, the prognosis is for the present much less unfavourable, but after the pleura is completely filled with pus instead of air, the patient still incurs the risk of a severe empyema, and, of course, under the best of circumstances, the prospects of ultimate recovery are doubtful.

*Duration and Termination.*—The duration of pneumothorax is not fixed. It may terminate fatally in a short period; in one case I witnessed a fatal termination in less than an hour, or it may last many months; in two cases I found the fatal termination not to occur until the lapse of fifteen and eighteen months; in the latter of these cases, the patient made two long voyages, and did full duty as a seaman. It is in this variety that the lesion is followed by empyema, and the possibility at least of recovery must be admitted.

*Treatment.*—The treatment of perforation of the pleura is extremely limited. The indications are to subdue the secondary inflammation, or rather to keep it within moderate bounds, and to relieve the pain. But as the patient is already much debilitated by previous disease, there is little to be done in the way of active treatment. Bleeding is quite inadmissible, but an occasional application of cups may be allowed, although with great reserve, and only in those cases in which the inflammatory excitement is very high. Blisters are much more frequently of benefit; in fact, they are the most certain remedies for checking the inflammatory action, and often relieve the pain; they should be applied to the affected side, near the seat of pain, which does not correspond in most cases with that of perforation. Besides blisters, the only remedy which promises much is an opiate; especially Dover's powders, given in doses sufficient to tranquillize the agitation of the patient; and if not to secure sound sleep, at least to relieve the incessant restlessness and suffering. This treatment I have long pursued in cases of pneumothorax, and it is nearly similar if not altogether identical with that recommended by Dr. Graves for the treatment of intestinal perforation in typhoid fever. The opiate should be continued for some days in a full dose, and in diminished quantity during the greater portion of the cases, discontinuing its employment when the oppression increases, or the digestive powers become much enfeebled.

The proper anti-phlogistic treatment of pleurisy is scarcely adapted to cases of pneumothorax; for as the cause is a permanent and mechanical one, it cannot be removed by antiphlogistic or alterative remedies, and, therefore, the progress of the secondary pleurisy cannot be retarded; the effect should be modified and the empyema, which is almost necessary to the cure of pneumothorax, should afterwards, if possible, be brought to a favourable issue.

The operation of paracentesis is sometimes allowable in two different stages of the disorder; to favour the escape of the gas, or the pus which is afterwards secreted. Immediately after the perforation the dyspnœa may suddenly become so great that immediate death is to feared; the side may then be punctured in the usual way, and the gas be allowed to escape; but as in this case, the subsequent dangers of the disease are certainly increased by exposing the cavity of the pleura so freely to the air, the operation cannot be justified except it be a measure of absolute necessity; at best, it relieves the patient only for a short time. In the cases of advanced empyema which follow pneumothorax, paracentesis may be performed where the oppression is extreme, and the intercostal spaces are much bulged out. The operation is, however, very far from being devoid of danger, for the free entrance of the air into the cavity tends to increase the inflammation, and to aggravate the hectic fever. The usual precautions should be carefully attended to after the operation.

